# The Untrustworthiness of the Reports of the Government in Relation to Investigations of Animal Diseases

**Published:** 1891-09

**Authors:** Frank S. Billings

**Affiliations:** Director of the Patho-Biological Laboratory, State University of Nebraska


### THE UNTRUSTWORTHINESS OF THE REPORTS OF THE GOVERNMENT IN RELATION TO INVESTIGATIONS OF ANIMAL DISEASES.

By Frank S. Billings, M. D.
Director of the Patho-Biological Laboratory,
State University of Nebraska.

This subject has been once before alluded to in an article published in The Journal by Dr. Spitzka, but I do not think the veterinary profession of this country or the original investigators in patho-biology in the world have any idea how gross are the errors, how absolutely false are many of the statements made in connection with the investigations of animal diseases, especially those of swine in the reports of the United States Department of Agriculture.

It is well known that I have constantly asserted the work of the Investigators of the Department of Agriculture on Swine Diseases, with the exception of that of Dr. Detmers, as published in the reports from 1880 to 1885, to be *unequivocally false*, and I now make bold to assert that there is no evidence that any actual investigations were ever made, and that all the evidence goes to show that the entire bacteriological material reported during that period was made up out of whole cloth.

### The Evidence.

The "micrococcus" as the specific germ of swine-plague first came into the imagination of the Government Investigators in 1880, that is, it is first reported in that year, and continued until the issue of the report of 1885 (published in the year 1886), when they did not consider a micrococcus to be the cause of all outbreaks of swine-plague. A quotation from the reports of 1883 and 1884 each, will answer all purposes for the whole series; especially is that of 1883 valuable as it has relation to inoculation also, a point in which the government is entirely at fault. From page 57, Report of 1883, I quote the following:

"Our investigations have shown that the plague is a non-recurrent fever, and that the germs might be cultivated; they have even proved that these germs may be made to lose their virulent qualities and produce a mild affection. Surely we have here sufficient evidence to show that a reliable vaccine might be easily prepared, if we carried our investigations but a little way further. If we had such a vaccine, if it were furnished in sufficient quantities and of a reliable strength, if it proved safe in the hands of the farmer, would not our problem be solved? Can we reasonably expect anything more or better for this disease?

M. Pasteur has recently confirmed our American investigations in a very complete manner. He shows that the disease is produced by a micrococcus, that it is non-recurrent; that the virus may be attenuated and protect from subsequent attacks, and he promises a vaccine by spring.

This should certainly inspire our people with confidence and it should incite our authorities to give the suffering pork-producers the full benefit of these discoveries at the earliest possible moment. There may be objections to vaccination, and I doubt not these are of certain importance; but with hog cholera already distributed over our whole hog-raising territory, these can have but little weight compared with the incalculable benefit that would be conferred by a practicable system of vaccination."

From the Report of 1884, page 221, also:

"In former reports details of experiments have been given which, if correctly stated, demonstrates beyond question that the microbe of swine-plague is a micrococcus. The experiments were made and accounts of them published in advance of those of M. Pasteur's and the evidence furnished was all that could reasonably be required to decide a scientific question of this kind."

My purpose now is to show once for all that the reports of the government have been, and still are, of such a decidedly inconsistent and contradictory character that no statement coming from that source can be taken with any confidence or relied upon unless so fully supported by collateral testimony, that that of the government need not be considered at all. Now what do the above quotations show to the general reader when taken by themselves?

1st. That the germ of swine-plague had been discovered by the government.

2d. That it was a micrococcus. To this assertion the government persistently held until sometime in the spring of 1886—that is, until the report of 1885 was issued.

3d. That tests had been made which warranted the government in promising the farmers that swine-plague could be prevented by inoculation.

4th. That the government found itself justly strengthened because M. Pasteur had confirmed all its work and also promised a virus for preventive inoculation.

Certainly the statements before us bore out exactly those conclusions. Now, as to M. Pasteur, he never saw a case of swine-plague for many years subsequent to 1883—if at all, as it was only discovered in France in 1887, and what is more, for the government, he studied Rouget and not swine-plague, and a "micrococcus" has been shown never to have had any causal connection with Rouget, so Pasteurs' confirmation was valueless and his promises of a virus also, so far as our American swine-plague is concerned. But we need not go to Pasteur to knock the whole government superstructure into pieces. It has a faculty of manufacturing "Boomerangs" never equalled by the most expert savages.

In the Report of the Department for 1890, (issued May last), may be read:

"*The first tests in this direction* (*for inoculation*) *were made early in 1886, soon after the hog-cholera bacillus had been discovered.*" Now honestly, and in the interests of truth only how can the above passage be made to confirm to the whole testimony contained in the reports from 1881 to 1886?

That the micrococcus was a fraud is self-evident, and never having been the cause of swine-plague it is equally evident that "our investigations (could not) have shown that the germs (micrococci) might be cultivated, or made to lose their virulent qualities and produce a mild affection."

In fact, nothing was "shown" then and nothing has been "shown" since.

In this JOURNAL, 1888, Vol. ix, page 149. may be read the following words from the government as to inoculation. "*We soon found that there was no indication for attenuating the virus for this purpose, because the strongest virus might be introduced hypodermically with impunity in considerable doses.*"

Now let us witness the government turn a sommersault. "In reducing the virulence or attenuating it, the following method was pursued." "*After the bacteria had been exposed to a high, unfavorable temperature* (110°-111°F.) *for more than 200 days.*"

Can any mortal human tell what that attenuation was done for in 1890—when in 1888, "the strongest virus could be injected with impunity in considerable doses"?

The reason for all these governmental psycopathological idiosyncrases is that at every point they have been completely beaten by other investigators.

In a recent letter to the editor of the "*Western Swine-herd,*" issue of July 1st., (who asked the government what it knew about inoculation), the government answered in a most peculiar manner. It referred the editor to one of its most gyroscopic productions, and endeavored to kill out the truth, published in the report of 1889, and cooly ignored its report of this year which as frequently contradicted that of 1889 as any of the examples quoted. A quotation from the letter mentioned follows:

> "Replying to your third question I would say that I have no means of knowing whether the wide prevalence of swine diseases was or was not due to the dissemination of the disease by the many attempts at prevention by inoculation. *I have no doubt but that the disease may be spread in that way,* and if, as is intimated in your question, there were many attempts made at prevention by inoculation, this may account for the unusual dissemination of the disease in the sections where inoculation was resorted to."

Perhaps no more contemptible or dastardly means could be taken to deceive the public than the above—or to endeavor to injure a great public interest.

1st. No such number of hogs were ever inoculated.

2d. Last year the loss in inoculated hogs, done on farms by the owners themselves, was not half of one per cent. in many thousands.

3d. Not a hog has ever been inoculated on farms already infected (except in one case). The fact that there were no deaths among the inoculated hogs, and that not an owner sent for inoculation (except a few very intelligent men of some years' experience) until the disease was all around him, speaks more emphatically for the truth and for inoculation than all such assertions.

But I need not say a word. The reader of the above quotation from the government will probably be afflicted with an acute attack of apoplexia when he turns to the Report of 1890, issued before the above letter was written, and sees the government recommending my system of inoculation, as the cheapest and best in spite of the terrible ravages of swine-plague it conjured up in its letter to the *Western Swine-herd.*

"This method of sub-cutaneous injections of culture liquids containing hog-cholera bacilli, while on the one hand *fraught with the possible danger* (*!!*) of scattering diseased germs where they do not originally exist, *is nevertheless the simplest and cheapest method* that can be devised for the vaccination of animals; these qualities of simplicity and cheapness are of vital importance in a question which has only a commercial aspect." With such contradictory publications frequently bearing his name, and always emanating from his department, can the Secretary of Agriculture for a moment think the governments of Europe will have an iota of confidence in his so-called "Meat Inspection" or any of his assertions regarding the same?

I leave the question at this point for the consideration of others.

